# “En la Lucha”: Strategies to Improve HIV Care for Puerto Ricans with Opioids Use Disorders

**DOI:** 10.1007/s10903-020-01091-6

**Published:** 2020-10-30

**Authors:** Miguel Muñoz-Laboy, Laura Bamford, Jose Benitez, Yaara Zisman-Ilani, Alexandra Ripkin, Laura del Castillo, Tracy Esteves-Camacho, Mario de la Cruz, Elby Katumkeeryil

**Affiliations:** 1 Department of Community Health and Social Medicine, School of Medicine, City University of New York, 160 Covenant Avenue, Suite 310, New York, NY 10031 USA; 2 Jonathan Lax Treatment Center, Medical Leadership, FIGHT Community Health Centers, Philadelphia, PA USA; 3 Prevention Point Philadelphia, Philadelphia, PA USA; 4 Department of Social and Behavioral Sciences, College of Public Health, Temple University, Philadelphia, PA USA; 5 FIGHT Community Health Centers, Philadelphia, PA USA

**Keywords:** HIV care continuum, Latinx, Opioid use disorder (OUD), Transnational populations, Injecting drug users

## Abstract

**Background:**

Clínica Bienestar is a comprehensive HIV primary care clinic for Spanish-speaking Latinx with opioids use disorders (OUD). This article describes the barriers and trajectories to HIV viral suppression for Puerto Ricans with a transnational profile and dual diagnoses (HIV and OUD), and the strategies applied to increase retention in care.

**Methods:**

Case study methodology was used to select two patient life histories that illustrate the most common pathways to success in reducing HIV viral load to undetectable and achieving OUD long-term recovery.

**Results and Discussion:**

Patients’ major challenges included: (1) Persistent migrating while seeking substance use treatment services with limited or no support from their sending and hosting communities; (2) Intersectional stigmas; (3) Untreated trauma; (4) Language and cultural barriers. Clínica Bienestar’s service model included ten strategies to retain patients in care (e.g., Case management to identify cases with high social isolation), six emerged as central to addressing transnational challenges.

## Resumen

**Antecedentes:**

La Clínica Bienestar es una clínica integral de atención primaria de VIH dirigida a hispanohablantes Latinx con diagnostico de dependencia al uso de opioids (DUO). El objetivo de este artículo es describir las barreras y las trayectorias para la supresión viral del VIH para puertorriqueños con un perfil transnacional y diagnósticos duales (VIH y DUO); y las estrategias utilizadas para aumentar la retención en cuidado medico.

**Métodos:**

La metodología de estudio de casos fue seleccionada para identificar dos historias de vida de pacientes que ilustren las vías mas frequentes para el éxito en reducir la carga viral del VIH a indetectable y lograr recuperación a largo plazo de la DUO.

**Resultados/Discusión:**

Los principales desafíos para los pacientes incluyeron: (1) Migración persistente en búsqueda de servicios de tratamiento de uso de sustancias con apoyo limitado o nulo de sus comunidades de envío y de acogida; (2) Estigmas interseccionales; (3) Trauma no tratado; (4) Barreras lingüísticas y culturales. El model de servicio de la Clínica Bienestar incluyó diez estrategias para retener pacientes en cuidado medico (por ejemplo, manejo de casos para identificar casos con alto aislamiento social), seis surgieron como elementos centrales para abordar los desafíos transnacionales.

## Background

Fewer than half of those with an opioid use disorder (OUD) receive treatment in the United States [1]. These unmet OUD treatment needs are more pronounced in Latinx communities [2–11]. In cities like Philadelphia, Pennsylvania, the zip codes with the highest concentrations of Latinx residents also have the highest opioid overdose-related deaths (an indicator of untreated OUD) and the highest annual HIV incidence rates over the past two decades [12, 13]. Latinx with untreated OUD have lower levels of retention in HIV care; and lower HIV viral suppression, and substance use treatment completion rates than other racial/ethnic groups, nationwide and locally in Philadelphia [14–16]. In order to decrease HIV viral loads at the population levels in Latinx communities, research examining detection, linkage and retention in HIV care is urgently needed, particularly amongst the subgroups with the highest incidence rates, such as Latinx with dual diagnoses of HIV and OUD.

When describing the service needs and challenges experienced by this subgroup, it is imperative to recognize the impact of transnationalism on their health seeking behaviors. Transnationalism describes the duality observed in many immigrant communities where community members may exhibit both, an allegiance and association with their “receiving” or hosting country, where they are making a new home and, simultaneously, their sending country or place of birth/origin [17]. The challenges faced by recent immigrants, who may need to access health services, to acculturate and adjust to new social norms, learn new vocabulary, and navigate the economic demands of daily life in a new place, can all contribute to difficult experiences accessing care. Cultural barriers and migration patterns such as the “air bridge” (i.e. the cyclical migratory patterns between the territory of Puerto Rico and the east coast of the contiguous USA), are challenges to cities like Philadelphia in the provision of HIV and OUD services for Latinx communities [18–22]. Philadelphia has experienced a 46% increase in its Latinx population in the past decade [23]. In response to this critical need for dual treatment for a transnational population, Prevention Point Philadelphia (PPP), Philadelphia FIGHT Community Health Centers (FIGHT), and Temple University’s School of Social Work established Clínica Bienestar (Spanish for “Wellness Clinic”) funded initially by the Special Projects of National Significance (SPNS) in December 2013. Clínica Bienestar is an HIV primary care clinic targeting Spanish-speaking or bilingual Puerto Rican adults (aged 18 + years) with previous or current injection drug use, or who meet criteria for OUD, who are newly diagnosed with HIV or who are not currently retained in HIV care (defined as being out of HIV care for at least 6 consecutive months in the previous two years– prior to intake) [24].

Clínica Bienestar’s model of service provision consisted of outpatient clinical services two days per week combined with HIV patient navigation, case management, OUD treatment, and recovery services throughout the week, as tailored to individual patient needs. The list of specific services has been published elsewhere [25]. The model has been effective at achieving HIV viral suppression with a statistically significant HIV viral suppression rate of 82.9% (95% confidence interval: 72.9%–93.1%) [25].

The objective of this article is to describe both the patient-level barriers identified and the contributing factors that influenced successful trajectory to HIV viral suppression for Puerto Ricans with dual diagnoses (HIV and OUD); including the strategies implemented in the Clínica Bienestar service model to improve retention in care. While this feature of persistent migration was not present for all patients enrolled in the larger SPNS demonstration project, all patients (prior to Clínica Bienestar) experienced language and cultural barriers to HIV and OUD care associated with their transnational backgrounds. Thus, the service model was guided by a transnational framework— one which identifies, acknowledges, and builds upon the connections that Latinx migrant populations may use to maintain ties to their sending countries/places of origin while living in the continental U.S [26]. Having a basic understanding of the extent that this demonstration project addressed the needs of a transnational sample of Latinx living with HIV who were out-of-care and dealing with co-occurring OUD and other substance use disorders could provide insights into other programs caring for similar populations.

## Theoretical Framework

### Conceptual Basis for Intervention

Based on more than two decades of organizational experiences since the onset of the HIV epidemic in Philadelphia, and the review of the scientific literature in HIV care continuum outcomes, the leadership of both organizations and the intervention designers decided to establish Clínica Bienestar as guided by three principles: (1) Co-location of services for transnational groups to increase utilization of HIV services by minimizing unnecessary navigation through complex health care systems (primary care services, HIV services, substance use disorder treatment) while decreasing duplicative costs to achieve similar health outcomes [27]. (2) Integrated dual treatment approach (IDDT), a well-documented, effective approach to mental health and substance use treatments, expanded to include HIV primary care services to substance use and mental health services for Latinx with dual HIV and OUD diagnoses [28–30]. (3) Harm-reduction, critical time (CTI) approach for transnational populations living with HIV focusing on people’s readiness for treatment and engagement in their own HIV and OUD treatment and care, and not requiring abstinence from substance-using before HIV treatment [31–35]. These above principles are relevant to understand the design of the overall SPNS intervention, as well as the tailoring of service delivery methods and strategies to address the needs of the patients who were also experiencing transnational challenges [36].

To conceptualize the experience of participants of Clínica Bienestar, we have to draw on the literature on intersectional stigmas, acculturative and minority stress. Before entering the doors of Clínica Bienestar, the population from where our participants emerged have experienced systemic forms of durable oppressions, well-documented to decrease the likelihood of engagement and retention in any form of health care service [37]. Because of the racial/class structure in the USA, Latinx are labeled into multiple combinations of minority statuses (racial, ethnic, gender expression, sexual orientation, social class) [37]. Latinx who use substances, living with HIV and OUD, experience additional forms of stigmatization manifested as systemic and interpersonal prejudice, rejection, discrimination, and violence against people who use substances and/or because of HIV status [19, 38–41]. Taken together, the additive effects of intersectional forms of stigma, in the absence of proper social support systems, result in experiencing high levels of minority and acculturative stress [37–42]. Minority stress refers to the continuum from distal to proximal stressors that derive from the personal embodiment of both internally and externally derived exclusionary processes, such as societal prejudice against people who use drugs, racial discrimination, anti-immigrant discrimination, and/or HIV stigmatization [39, 43–48]. Acculturative stress refers to the pressures of learning a new language, the reactions to language-related micro-aggressions, balancing differing cultural values, and having to broker between American and the culture of origin ways of daily living [49].

Minority and acculturative stress further delay HIV and OUD care because these are demonstrated predictors of anxiety, depressive, and substance use disorders severity, suicidal ideation, and low utilization and retention in HIV and substance use treatment and care services [43, 49–57]. The following analysis will provide the strategies that were used to mitigate the additive effects of intersectional stigmas, minority stress and acculturative stress for a transnational Latinx population with dual diagnosis of HIV and OUD.

## Methods

### Participants

From September 2013 to December 2017, 70 individuals consented and enrolled (average age = 43.2 years; 81.4% males; 0% transgender) into the Clínica Bienestar SPNS project. The majority were born in the US territory of Puerto Rico (77.1%). Between 10 to 15% of the sample at any given month during the longitudinal study was incarcerated at the county jail. 41.8% reported being homeless or being in an unstable housing situation most of the last month. Most participants (79.2%) were single (not cohabitating, not married). Most (88.5%) participants were out of HIV care before linkage to Clínica Bienestar, and the remaining were newly diagnosed with HIV (11.4%). The top five untreated physical health issues identified at baseline clinical assessment were: (1) hepatitis C virus (64.4%), (2) obesity/overweight/underweight (42.2%); (3) Hypertension, pulmonary hypertension (30.1%), (4) Gastroesophageal Reflux Disease, GERD (26.7%), and, (5) Asthma (15.6%). The top five untreated mental health issues identified at baseline clinical assessment were: (1) tobacco use disorder (82.2%), (2) cocaine, stimulant, polydrug use disorders (64.4%), (3) depressive disorders (60.0%), (4) generalized anxiety disorders (33.3%), and, panic attack disorders (11.1%). Close to a quarter of participants had severe neuropathy at baseline (22.2%). Each patient received a personalized treatment plan for each co-occurring physical health or mental health concerns/conditions.

To present detailed descriptions of the experiences and trajectories in accessing and obstacles to HIV and OUD care; and the effects of the strategies used by Clínica Bienestar, we used Cresswell’s and Yin’s approaches to case study methodology, specifically confirming/disconfirming case study methodology (described below) [58–61]. Drawing on the principle of salutogenesis (briefly defined as a medical approach focusing on factors that support human health, resilience, coping, and well-being, rather than on factors that cause disease), our analytical focus was on cases that successfully reduced their viral load to undetectable and were retained in HIV/OUD care for 24 months [62].

### Data Collection

The qualitative process evaluation design of this SPNS demonstration project consisted of using case study methodology of life history interviews with the first ten individuals who completed the 24 months of the longitudinal study. To be considered for the interviews potential participants had to be HIV virally suppressed for at least one year, and currently in treatment and/or recovery for their substance use disorders. Using the aforementioned criteria to develop a list of potential participants, individuals were contacted in the order of when they had completed the required 24 month period. The first ten patients who completed the 24 months of study participation were interviewed.

Drawing from the literature on biography research, a life history on HIV and substance use in this study refers to the construction of a chronological narrative of the events related to HIV acquisition, living with HIV, substance use trajectory, and experiences in care in the life of an individual; and the perceptions, reactions, meanings, feelings, thoughts, and life events that surround those experiences [63, 64].

The life history consisted of one initial open-ended, in-depth interview of 45 to 90 min; followed by a second interview, within two weeks, to clarify themes or inconsistencies in the narrative. Participants received monetary compensation of USD$25 for their time. The interviews were conducted in Spanish, English, or both, depending on the participant’s choice. The interviews were audio-recorded and transcribed within two weeks from the day of the interview by a subcontracted independent transcription company. The life history interviews were covered under the consent for the Clínica Bienestar SPNS project, however, consent procedures were reviewed with each participant before the interview. Interviews were conducted by a highly trained life history interviewer, who had more than 5 years of interviewing experience, and their only role on the project was as an interviewer for this specific research activity.

The life history interview guide consisted of five primary elicitation themes: (1) migration trajectory to Philadelphia; (2) HIV diagnosis and trajectory to engagement in care at Clínica Bienestar Philadelphia; (3) Substance use trajectory into current treatment and recovery; (4) Mapping of kinship and community support systems; (5) Services received for the past 24 months at Clínica Bienestar. The guide was divided into primary questions and probing statements. Theme #1, the migration trajectory, served as the core element to build the narrative of the individual by asking questions about their journey to and arrival in Philadelphia. Then, the other thematic questions (#2–5) were asked with additional probes in relationship to the individual’s migration history. This process allowed for topics related to their migration journey to be revisited throughout the interview and for a fuller narrative exploring their migration trajectory to be developed. Apparent initial inconsistencies in any participant’s narrative were revisited during the interview with additional probes or in the follow-up interview if needed. This study was approved by the Institutional Review Boards of Philadelphia FIGHT Community Health Centers and Temple University.

### Data Analysis

The first author, along with an independent coder, coded the life histories according to the theoretical framework: (1) family composition; (2) migration trajectory; (2) transnational life in Philadelphia; (3) HIV treatment and care trajectory; (4) substance use treatment and care trajectory; (5) acculturation processes; (6) acculturative stress experiences; (7) minority stress experiences; and, (8) stigma experiences. The interviews were coded using Atlas.ti software. To begin analysis of the interview data, the team utilized Cresswell and Yin’s case study methodology. This methodology is characterized by first identifying cases that illustrate recurrent themes across all the cases [58–61]. The team sorted through the ten cases and selected two cases that best illustrated all the five theoretical thematic codes. Upon completion of all coding and team discussions of the narrative data, it was clear that not one case study represented all the histories within the group. Thus, this qualitative analysis focuses on two of the 10 life histories conducted as part of the evaluation of Clínica Bienestar and its service model for this SPNS initiative: (1) Ms. Gloria, pseudonym, self-identified as a woman, uses the pronouns, she, her, hers; was 49 years old at the time of the interview. (2) Mr. Tito, pseudonym, self-identified as a man, uses the pronouns, he, him, his; was 47 years old at the time of the interview. Ms. Gloria and Mr. Tito were selected because: (a) they represented opposite poles in their level of social assets/resources available in the context of transnationalism and accessing care and treatment services; (b) each case represented gradients of success in managing their substance use disorders, and; (c) they offered differing gender and sexual orientation perspectives from cis-gender heterosexual man (Mr. Tito) to cis-gender lesbian woman (Ms. Gloria). These two cases do not represent the total number of trajectories or the full spectrum of experiences into HIV and substance use treatments among study participants but rather provide an example of the variety of experiences identified across the entire sample [59–61].

## Results

### Case Study #1: Life History of Ms. Gloria

#### Migration Trajectory

The first participant selected as a case study, Ms. Gloria, grew up traveling back and forth between Puerto Rico and Philadelphia. After beginning to engage in substance use at age 14, Gloria agreed with her family to go to Philadelphia to seek treatment for what she called her “vicio” (Spanish for vice). She was 23 years old. Her primary or sending home, however, was Puerto Rico, until that year.

In Philadelphia, she stayed with her aunt. At the end of that year, after turning 24 years old, she became homeless. Her aunt expelled her from her house, when Gloria did not complete her outpatient substance use treatment program. She began to use heroin and cocaine again, her primary drugs of choice.

Her oldest daughter was born in Puerto Rico when Gloria was 16 years old. By her mid-twenties, she gave custody of her three daughters to her mother, who was raised at first in Puerto Rico and over the past decade in Philadelphia. Gloria is now a grandmother of a 7-month-old baby. Currently, almost all of her family lives in Philadelphia.

During the four years before the interview, Gloria traveled with less frequency between Philadelphia and Puerto Rico, as she used to do before engagement in Clínica Bienestar. With regards to her migration pattern, she said:

**Table:** 

Yendo y vieniendo a la isla no me ayudo	Going back and forth to the Island, didn’t help me

During the winter months, she expressed her desire to return for long-periods to Puerto Rico, particularly to celebrate Christmas and New Year’s. Her level of contact with her kinship network in Puerto Rico, however, varied throughout the past two years. She expressed feeling isolated from her family in Philadelphia and excluded from her family in Puerto Rico, a dual sense of loss associated with each place she has called home in her transnational experience Gloria prefers speaking Spanish over English; watches news and shows in Spanish; and rarely speaks English except when she has to. This discomfort communicating in English has at times presented a barrier for her when trying to articulate her service needs or access care in Philadelphia.

#### Substance use Treatment Trajectory

Gloria has had no support from her family on her substance use treatment trajectory. Gloria’s family communicates with her via text only, not in person, or by phone.

**Table:** 

No, mi familia no quieren cuenta conmigo por ahora. Porque ellos no creen que yo puedo quedarme limpia. Ellos dicen que es por un ratito y vuelvo a lo mismo. Pues yo estoy poco a poco demostrándoles. Yo llevo ya pa' un año!	No, my family wants nothing to do with me. Because they do not believe that I can stay clean. They say that it is just for a little bit and then I would turn back to the same. However, I am showing them little by little. It has been a year!

Gloria has been using substances since she was an adolescent. She does not provide details of those early experiences. She rather focuses on her trajectory through substance use treatment programs. She tried multiple programs without completing them. The longest period that she has gone without using substances was three years in her early twenties before relocating to the United States mainland. She thought that moving to Philadelphia would allow her to “regain control” over her substance use. Seeking and buying heroin and cocaine for herself and her girlfriend, who later became her wife, consumed most of Gloria’s time. At the height of her use, they rarely had sex together except when they were doing it in exchange for money or drugs.

She tried suboxone on multiple occasions. It did not work, she thinks, because she was not truly invested.

**Table:** 

Yo todavía estaba en esa actitud de síguelo usando. Yo pensaba que todavía tenía una carrera más en mí… yo ahora tengo artritis crónica. Tengo Lupus, tengo neuropathy, tengo – padezco de seizures. Tengo asma, tengo un heart murmur. Tengo – soy major depression, bipolar. Tengo un montón de condiciones médicas	I was still in that attitude of keep using. I thought I would have one more run in me… now I have chronic arthritis. I have lupus, neuropathy, and also suffer from seizures. I have asthma and heart murmur. I have – I am, major depression, bipolar. I have a lot of medical conditions

Gloria sees the emergence of these co-morbidities as alerts to address her dependence on substances. Over the last year, she has not used substances. Currently, Gloria is on daily methadone, and receiving psycho-social services for her substance use disorder at Clínica Bienestar. She has not had cravings. Gloria explained how she walks to places that sell heroin or cocaine and she feels nothing. People give her “gifts” of substances that she has passed along to other users. “This has shown me how serious I am about my recovery,” she expressed.

#### HIV Diagnosis

In 2002, when she was in her late 20 s, Gloria was diagnosed with HIV while in a correctional facility in Puerto Rico. Back then, she said, there were no treatments available. People with HIV were dying constantly. The only way she could get HIV medications was inside correctional facilities. She had been imprisoned on drug-related charges both in Puerto Rico and Pennsylvania. Outside prisons and jails, HIV medications were too expensive.

She is proud of achieving HIV viral suppression.

**Table:** 

Estoy undetectable. That's the word. Undetectable. Y ahora a mismito mi esposa está en la calle todavía utilizando. Yo llevo con ella ocho años. Hace como un año y pico que yo no estoy con ella	I am undetectable. That’s the word. Undetectable. Right now my wife is on the streets using. I have been with her for eight years. I have not been with her for more than a year now

Gloria’s wife was her closest source of social support until a year ago when Gloria moved out of her wife’s place. She has not dated anyone else since then. Isolated from her family because of stigma and with few other sources of social support due to her transnational migration, dating was a form of support for her, for not feeling lonely. Yet, her dating often led for Gloria to relapse into using substances again. For her, going for more than 12 months without using drugs has been a major accomplishment that to some extent she adjudicated to living separately from her wife, who is still actively using opioids and other substances. This commitment to remaining sober is further evidence of the behavior and attitude change that she self-reported earlier in the transcript.

#### Intersectionality and Discrimination

In her experience, most of the discrimination she has suffered is because of multiple combinations or permutations of discriminatory rejections because of her homelessness, HIV status, drug use and/or her sexual orientation as a lesbian.

**Table:** 

En los half-way, te tratan como una ignorante, hechándoselas, dejándote saber quién manda	In the half-way houses, they treated you as if you were ignorant, condescending and often showing off their power

Housing discrimination has been a challenge for Gloria. From 2014 to 2018, she was homeless or living in between half-way-houses, which often only allow people to stay for a maximum of between 60 and 90 days. In the instances when she had accumulated enough money to find herself a place to rent, potential renters expressed to her that they did not trust that she would have a stable source of income to pay rent consistently. It is in those instances that she would feel her depression worsening. Her HIV status made her feel even more depressed and would lead her to instances of catastrophizing her health conditions.

Gloria has also experienced discrimination by health care providers and in social services agencies because of her HIV status and her sexual orientation.

**Table:** 

Cuando uno va al hospital y uno dice, "Mira, yo soy HIV, soy una adicta", ahí rápidamente te ponen como un red flag…Estás sellada, tú sabes, estás – una estampa. Mira, esta es una ____. Esta tiene HIV. Sí hay maneras de – y que – que también los doctores cuando te suben, que te admiten y todo, ellos se paran a hablar de tu condición cuando pacientes van por aquí y alla	When I went to the hospital, and one says: “I am HIV, I am an addict,” quickly they put a red flag on you. Look, she is a blank. She has HIV. Yes, there are ways in which also doctors when they get you up to see them, and admit you, and they stand to talk about your condition in front of other patients coming in and out

Yet, the most powerful experiences of HIV related discrimination came from her family.

**Table:** 

No respeto para nadie y es triste. Porque hay personas que no son fuertes y se han quitado la vida por eso, por la discriminación. Como yo cuando mi familia lo supo, ellos limpiaban el toilet con – que yo me metí a bañar, cuando yo salía, ellos se – mi hermana se ponía guantes, cogía cloro. Le decía a mi sobrina que cuando yo entrara al baño, que no entraran detrás de mí, que esperara – fuera a donde ella, le dijera, mira, yo usé el baño, para ya limpiarlo. Hasta la ducha y todo. Yo tenía una cuchara y un vaso para mí	There is no respect for anyone and it is sad. Because there are people that are not strong and they take their lives, because of discrimination. Like me. When my family learned about it [HIV status], they start cleaning the toilet – I would go to the bathroom and shower, and when I would leave, they – my sister would put on gloves, and take the bathroom with bleach. She would tell my niece that when I was going to the bathroom not to come after me and to wait—to come and tell her so that she could come and clean it. Even the shower. I had my own spoon and cup

#### Engagement in HIV Care

Gloria was living under the bridge in Kensington, Philadelphia, PA, when one of the outreach workers of Clínica Bienestar approached her. The outreach worker was trained in addressing cultural barriers and using trauma-informed care approaches. The young woman outreach worker spoke to Gloria about the syringe service program. Gloria expressed how the outreach worker’s welcoming and respectful approach made her feel safe. The non-judgmental invitation was distinct from previous stigma filled and negative experiences Gloria had when trying to access services in the past, this had a direct impact on her engagement level upon entry into care and her retention in care and treatment services.

**Table:** 

Mi salud, mi salud ha mejorado. Estoy indetectable. He aumentado de peso. Cumplo con todo lo que tengo que cumplir. Sí, todo, gracias a Dios a mejorado mi salud, mi forma de vivir. Ahora está un poquito más estable de lo que estaba antes – antes que estaba aquí y allá. Ahora estoy en una media casa. Voy a mis citas. Hago grupo de tres veces a la semana. Voy a Comhar a ver el psiquiatra y a ver mi consejero, psicólogo ahí en “Lindos” hospital. Aquí deben de – ya que han luchado y han podido lograr muchísimo ustedes. Luchar para que abran medias casas o shelters de mujeres, que no hay	My health, my health has become better. I am undetectable. I have gained weigh. I do everything that I am ought to do. Yes, everything, thanks to God my health has become better, and so my lifestyle. It is now a Little bit more stable than it was before. I am in a half-way house. I have my appointments. I go to a group three times a week. I go to see my psychiatrist and psychological counseling whenever the appointments are in “Lindos” hospital [pseudonym]. And here, fighting, and you and I have achieved a lot. I am in the fight now to open half-way houses for women, and women’s shelters because there are none [in Philadelphia]

Gloria began to attend the sterile syringes exchange program weekly. Two weeks later, Gloria progressed into seeking services beyond the syringe service program ranging from lunches, clothes, McDonald cards, wound care, and enrolling in Clínica Bienestar Philadelphia (see Table 1 Clínica Bienestar strategies). Gloria had a full HIV primary care visit within three days of accepting becoming part of Clínica Bienestar. She was connected to a near-peer patient navigator (briefly defined as a person living with HIV of similar demographics to the patient who had also been through the process of HIV and substance recovery treatments) to show her through the navigation of HIV, substance use and mental health care services offer within the space of Clínica Bienestar and through referrals. Over the next two years, Gloria participated in intensive case management given her high social isolation and severity of her substance use disorder. She participated, and became one of the leaders of the women’s support group.

**Table 1:** Transnational challenges and strategies in HIV and substance use continua of care, Clinica Bienestar, Philadelphia, 2013–2018

Challenges associated with transnational experiences	Nuances and differences between cases regarding challenge	Strategies to meet clinic participants needs
(1) Migrating to Philadelphia seeking substance use treatment services with limited or no support from their sending and hosting communities	Having close family network connections that did not necessarily translated into social support When substance use has been a multigenerational issue (Mr. Tito’s case), it increases likelihood of early onset of OUD, and low treatment program completion Strong held homophobic family beliefs, family beliefs that substance use is a failure of character, and poor family understanding substance use treatment produced further internalized stigmatization (Ms. Cariega)	Case management to identify cases with high social isolation, limited social support networks or challenging familial relationships, and using motivational interviewing techniques to design patient/client-centered strategies to manage the lack of family-based social support Linkage to social support groups within the spaces of PPP and FIGHT *Mitigating effects of the above in addressing challenges from transnational experience:* (a) Increased social connectivity; (b) Increased informational, emotional, and instrumental support for patients
(2) Stigmatizing experiences including rejections because of their accents, being people who use substances, being people living with HIV, having criminal records, and, not having steady sources of income	Both cases had limited knowledge of their civil and legal rights, leading to housing issues, recurrent arrests; and recurrent sentences for criminal charges Both cases illustrate the perverse incentive of having access to HIV medications through imprisonment, and no access while not incarcerated Ms. Cariega experienced additional discrimination because of being openly lesbian	Participants were exposed to educational materials on their rights Legal aid services were provided onsite to assist on health-harming legal needs and referral to legal aid representation for criminal defense Staff conducted strategic meeting to reinforce case management strategies to assist participants with housing, food, and supportive services *Mitigating effects of the above in addressing challenges from transnational experience:* (a) Decreased barriers to care from social determinants of health; (b) Increased patients management of stigmatizing experiences
(3) Trauma associated with the transnational experience of people with severe substance use disorders including interpersonal violence and sexual coercion together with fear of law enforcement involvement	Both cases documented multiple instances of witnessing violent deaths, overdose related deaths, street-violent related injuries, and being recipients of interpersonal violence, and, gender-based violence	Showing respect and trauma-informed care, timely services in one location provided the backdrop for addressing trauma in behavioral health services Staff was trained on trauma-informed care for issues affecting transnational populations *Mitigating effects of the above in addressing challenges from transnational experience:* (a) Empowered participants in engaging in their HIV and OUD care; (b) Initiated processes for healing and recovering from untreated traumas
(4) Language and cultural barriers, both cases did not know how to navigate highly complex mental health and health care systems	Ms. Cariega met criteria at baseline assessment for borderline personality disorder and depressive disorder Mr. Bracamonte met criteria at baseline assessment for persistent depressive disorder Depressive and anxiety disorders were difficult conditions to address in an HIV primary care setting for two reasons: (1) in public mental health care settings in the Philadelphia area prioritize the treatment of patients experiencing psychosis, suicide attempts, or major impairments; and, (2) Latinx are more likely to feel stigmatized in accessing mental health services and have low uptake and high stigmatization of anti- anxiety and depressive medications	Near-peer patient navigation to show and support participants through the navigation of mental and health care systems Monthly staff meetings were dedicated to evaluate and reassess the organizational environment, cultural sensitivity and implicit biases to promote a nurturing, trusting, safe and brave patient/client-provider relational space Colocation of services and timely responses to patients needs could have potentially minimized discriminatory encounters within the health system *Mitigating effects of the above in addressing challenges from transnational experience:* (a) Empowered participants in navigating in their HIV and OUD care, and treatment of other co-morbidities; (b) Initiated processes for connecting and retaining patients in care; (c) Facilitated critical time interventions to addressed acute and severe health and mental health situations of patients

### Case study #2: Life history of Mr. Tito

#### Migration Trajectory

Born in the Bronx, New York, Mr. Tito began his transnational life at three years old, moving to New Jersey, then back and forth between Pennsylvania and Puerto Rico. His parents would move to Puerto Rico and stay there for years. At 15 years of age, Tito and his brother were sent from Puerto Rico to rural Pennsylvania to live with their aunt. Less than a year later, both were arrested for their first time on drug possession and trespassing charges.

During the four years before the interview, Tito traveled once a year or once every two years to Puerto Rico but did not stay for periods longer than a month. He maintains close contact by phone and through social media with some of his family members in Puerto Rico. He expressed concern for friends or other “usuarios” (his word in Spanish for substance users) who recently arrived and “no conocen las calles” (his phrase in Spanish for not knowing the streets or being aware of drug culture in New York City). Unlike Gloria, he feels supported by his family in Philadelphia and Puerto Rico. Similar to Gloria, Tito also prefers speaking and writing in Spanish over English; he watches news and shows in Spanish; and rarely speaks English. This discomfort communicating in English has at times presented a barrier for him when trying to articulate his service needs or access care in Philadelphia.

#### HIV Trajectory

Tito tried cocaine at 15 years of age, and at “al principio todo es color de rosa” (Spanish idiom for everything is wonderful at first) suggesting that his substance use was not as wonderful as he aged. Months after trying cocaine, he started smoking marijuana which he continued doing from age 15 until his late 30 s. At 17 years old, he experimented with heroin and described in detail that first high on heroin. In summary, the high was more intense and superior than cocaine. He began snorting heroin regularly. Later that year, he was arrested and sent to a county jail in Pennsylvania. Contraband heroin was the available drug inside that correctional facility.

**Table:** 

Pero la cosa es que la cantidad que te daban no era suficiente para tú metértela por la nariz y emboyarte, sentir el arrebato. Pues me puyé, eso por la vena. Y ya – a la que le perdí el miedo, cuando salí de la cárcel, las veces que lo hacía, lo hacía haciendo spitballs, perico y heroína ligados. Y esa era – estuve un tiempo así. Me cuidaba de dónde me puyaba que no lo viera la gente. Uno pensando que la gente no se va a dar cuenta, pero la gente se da cuenta rápido. Uno se está engañando uno mismo	But the thing is that the amount of you got wasn’t enough to get you high through snorting. So I injected myself, in the vein. And that was it – at the moment I lost the fear to it, when I left the jail, I did it sometimes speedballs (heroin and cocaine combined). I was doing that for a while. I used to be careful where I injected so people would not see the marks. One [continues doing it], thinking that people would not know, people know really fast. One is lying to oneself

In 2005, he went to jail again for 2 months for possession of paraphernalia. “No soy un criminal o una persona que hace daño,” (Spanish for I am not a criminal or a person that causes harm). “Boberias” (Tito’s word for stupid, silly things) have gotten him imprisoned including trespassing, accumulated unpaid citations, charges for small amounts of drug and paraphernalia possession. It was in jail in 2005 that he was diagnosed with HIV. He thinks that he didn’t acquire HIV from a contaminated needle or sharing cookers but rather through sex. He learned how to inject and use clean needles from his relatives. Furthermore, he remembers rarely using condoms in his teens and twenties.

#### Intersectionality and Discrimination

In his family, and neighborhood environment he doesn’t remember being discriminated against for having been incarcerated, using substances or being Puerto Rican. However, Tito described frequently being discriminated against when trying to get a job because of his criminal record; and several instances of racial discrimination. Having HIV added a new layer of discriminatory experiences for Tito. His sister and two of his nephews know about his HIV status. Two years before arriving at Clínica Bienestar, he needed a place to stay so a friend offered to take him in. Tito disclosed his HIV status to him. His friend made Tito use disposable plates, cups, and utensils. Since then, he doesn’t disclose his HIV status unless the other person “really needs to know.”

Tito has also experienced HIV related discrimination from doctors. In one of the examples, Tito entered the physician’s office, who extended his hand to greet and receive him. At the moment of leaving, the doctor would not respond to Tito’s extended hand to say good-bye. Tito’s attribution is that this change in salutation tone had to do with hearing about his HIV status, medical, and, substance use history. Tito did not return to this provider.

#### Substance use Treatment Trajectory

Over the 2005 to 2014 period, Tito attended more than ten substance use treatment programs. Tito began treatment for substance use for the 11th time while he was beginning to receive syringe exchange services at PPP. He woke up in his bedroom one morning in October of 2015, with a rope tied around his neck, of which the other end was just lying on the floor next to him. He had no recollection of how he or the rope got there. He called his sister scared. She brought him to the hospital for detoxing and treatment for substance use. Shortly after completing his initial stage of treatment, Tito’s nephew died from a lethal overdose. He recounted this event as devastating. His nephew died with the wave of friends and “fulanos” (Spanish for acquaintances) who began to die rapidly in Philadelphia from fentanyl and heroin-related overdoses.

**Table:** 

Mira, fulano de tal se murió	Look! So-and-so died

It is in this moment in his life, Tito expressed, that he began to understand his depression, his sadness, the emotional and the physical pains he was experiencing as a result of his substance use, and his recurrent suicidal ideations. He was not prescribed antidepressants. His depression has continued until today, yet, he has not been able to stay in psycho-social therapy for his depression. Nor is he interested in taking anti-depressants. Throughout Tito’s account of this period, he mentioned how God and the Virgin Mary helped him from dying or killing himself. From his perspective, his faith and their mercy kept him alive.

#### Engagement in Comprehensive HIV Primary Care

It is through the syringe exchange program mobile outreach that Tito was recruited into Clínica Bienestar in 2014. At that point, he had not seen a non-emergency medical provider for more than 10 years. He was not taking HIV treatment medications.

**Table:** 

Cuando uno lleva más de 2 o 3 días sin bañarse uno apesta. Y aquí nunca nadie me hizo sentir mal porque yo no me había bañado. De la misma forma que me trataron el primer día que vine, hasta hoy día ha sido la misma. Nunca nadie me ha hecho sentir mal, más que el señor este	When one is without taking a shower for two to three days, one stinks. But here no one ever made me feel bad for not having showered. The way they treated me on that first day to today, it has been the same. Never, anyone has made me feel bad. They treat with “Señor” blank

The literal translation from Spanish to English of the word “señor” is “Sir” or “Mister,” but neither of those captures the level of respect that the words “señor” or “señora” carry. The non-judgmental invitation was distinct from previous stigma filled and negative experiences Tito had encountered when trying to access services in the past. Also, like Gloria, this respect for the individual and the recognition of their personhood had a direct impact on his engagement level upon entry into care and his sustained retention in care and treatment services. Señor Tito “Bracamonte” (Pseudonymn) was using heroin and cocaine during his first year of comprehensive HIV treatment.

**Table:** 

Porque todavía mientras estaba aquí, y por más que tú digas que no, de la forma que el vicio lo agarra a uno, ahora puedo darme cuenta de cómo me tenía agarrado, que me dominaba, yo era esclavo de la droga. En otras palabras, sí, era una esclavitud; porque al principio era todo color de rosa, pero ya a lo último solo lo hacía para no enfermarme, porque ya me podía meter 40 bolsas, y sí me agarraba. Y eso sigue creciendo y sigue creciendo y sigue creciendo	While I was here at first… no matter what you tell yourself, the way the vice grabs you, no I can see how it had me, that it dominated me, I was a slave to the drug. In other words, it was slavery; at first everything was wonderful, but at the end you just do it so you don’t get sick, because I could inject 40 bags a day, and it grabs you. And then it keeps growing and growing

“Estar solo no es facil” (Spanish for being alone is not easy), is how Tito described one of the major stressors of having HIV. Disclosing his HIV status to intimate partners was and is something that he finds more difficult than using condoms. Here the stigma that was feared from previous experiences went beyond solely being about his phyical appearance and housing status, there was also the fear of stigma associated with one’s HIV status. The Bienestar model of service provision, including case management and trauma-informed care and clinical services had a positive impact on Tito’s level of engagement and retention in HIV care and substance use recovery treatment. He has had sexual partners throughout his life, although not at the time of the interview. Tito has achieved HIV viral load suppression and maintained it for the past 18 months.

#### Strategies used by Clínica Bienestar to Address Patients’ Transnational Challenges

Table 1 lists the transnational challenges in-common between the above two case studies of Gloria and Tito, and also the nuances and differences between cases regarding each challenge. There were four in-common transnational challenges: (1) Migrating to Philadelphia seeking substance use treatment services with limited or no support from their sending and hosting communities; (2) Rejections because of their accents, being people who use substances, being people living with HIV, having criminal records, and, not having steady sources of income; (3) Trauma associated with the transnational experience of people with severe substance use disorders; (4) Language and cultural barriers, both cases did not know how to navigate highly complex mental health and health care systems. Ten strategies were used by Clínica Bienestar to meet clinic participants' needs (see Table 1). Six of these strategies emerged as most influential and important to consider when providing care and services for HIV and OUD to the transnational Latinx population that travels between Puerto Rico and the United States mainland. They are: (1) Case management to identify cases with high social isolation, limited social support networks, or challenging familial relationships; (2) Linkages to social support groups within the spaces of PPP and FIGHT; (3) Staff conducted strategic meetings to reinforce case management strategies to assist participants with housing, food, and supportive services; (4) Showing respect and offering trauma-informed care, and timely services in one location; (5) Staff was trained on trauma-informed care for issues affecting transnational populations to increase their service delivery capacity; and (6) Near-Peer patient navigation to show respect and support participants through the navigation of mental and clinical health care systems.

Along with case management, both Gloria and Tito identified that attending social support groups and being paired with Near-Peer patient navigators, who had also been through the process of substance recovery treatment themselves, had a positive influence on their engagement and retention in care and services. These services were also identified as positive contributors to successful engagement in care and services by the majority of participants interviewed in this sample. Additional research may yield deeper understanding of the degree to which each of the above-named strategies influence engagement or retention in care and reveal causal relationships or interdependence between individual strategies as drivers for patients’ uptake of behavior change.

Dealing with the stressors produced by intersectional stigmas, addressing traumas connected to transnational migration, and managing experiences of discrimination when accessing clinical services, are all project objectives interwoven throughout the strategies listed in Table 1. The organizational and staff capacity and history of dealing with substance-using populations of PPP and FIGHT facilitated a flexible implementation of strategies tailored towards the needs of each patient. Furthermore, three-quarters of the staff members had personal experiences as members of transnational communities themselves (i.e. Puerto Rican born, first-generation Puerto Rican, recent migrants, first-generation members of Latinx or other migrant communities) which contributed to their ability to provide care and services for the participants in this sample of the first ten out of seventy who completed the 24 months SPNS longitudinal demonstration project.

## Discussion

The life histories of Ms. Gloria and Mr. Tito illustrate the social profiles of transnational Puerto Ricans dealing with dual diagnoses of HIV and OUD (and other substance use disorders). In their social profiles, we see persistent and on-going movement before engagement in treatment at Clínica Bienestar between the territory of Puerto Rico and the states of New York, New Jersey, and Pennsylvania. Clínica Bienestar offered culturally-appropriate services tailored for a transnational Puerto Rican audience seeking safe spaces to receive services. In these cases, as in the rest of the life histories, they minimized their traveling, on their own volition during the first twelve months of receiving out-of-patient services in Clínica Bienestar as a way of prioritizing and demonstrating a commitment to their engagement in care and recovery treatment. Thus, the case studies above suggest that providing a transnational-cultural space, that may satisfy some needs of cultural connectedness to address social isolation, together with a safe organizational space that directly supports the management of the multiple stigmas and the stressors associated with HIV and OUD, might have had positive effects in increasing retention in HIV/OUD treatment and care.

The examination of the life histories of successful case studies from Clínica Bienestar reaffirms the point that epidemics driven by structural factors, such as the HIV and OUD, require structural responses such as the strategies used in Clínica Bienestar. The trajectories into HIV services for Ms. Gloria and Mr. Tito illustrate the trajectory of most patients/clients of Clínica Bienestar who had been diagnosed several years ago but rarely or never accessed HIV medical services. For a population, such as the cases presented, that has been lost to care in the medical and health and social services systems in both Puerto Rico and Pennsylvania, the co-location of services is critical.

We titled this manuscript, “En la lucha,” Spanish for in the fight, a phrase that Ms. Gloria and other patients/clients in the clinic used to refer to the daily fight to manage their substance use disorder and adhere to HIV medications. As health professionals serving transnational populations of PLWH with co-occurring substance use disorders requires, joining the fight for effective use of resources, for the development of cost-effective, sustainable, multi-level interventions, such as Clínica Bienestar Philadelphia, that can bring health and hope to individuals such as Ms. Gloria, Mr. Tito, their families, and transnational medically-underserved communities.
